# From reference-genome prediction to causal generalization: sequence-to-function models for plant regulatory genomics

**DOI:** 10.3389/fpls.2026.1968878

**Published:** 2026-09-17

**Authors:** Mingfang Zhan, Ruigang Wu

**Affiliations:** 1 College of Life Sciences, Wuchang University of Technology, Wuhan, Hubei, China; 2 School of Landscape and Ecological Engineering, Hebei University of Engineering, Handan, China

**Keywords:** cis-regulatory code, cis-regulatory elements, crop improvement, genome editing, machine learning, pan-genome, plant genomics, sequence-to-function models

## Abstract

Sequence-to-function (seq2func) models predict molecular regulatory outputs from DNA sequence and can prioritize non-coding variants, annotate cis-regulatory elements (CREs), and design regulatory sequences. Although accuracy has improved, most models are trained and evaluated on observational data from one or a few reference genomes. Their performance may therefore not translate to natural haplotypes, structural variants, tissues, developmental stages, environments, or species. These limitations are especially important in plants, where pan-genomic diversity, transposable elements, polyploidy, long-range regulation, and genotype-by-environment interactions shape regulatory landscapes. This Mini Review summarizes seq2func modelling in plant regulatory genomics, discusses plant-specific challenges and the complementary value of observational and perturbation assays, and proposes evaluation frameworks based on biologically meaningful distribution shifts. We further outline how pan-genomes, multi-omics, genome editing, reporter assays, active learning, and continual learning could support closed-loop experimental–computational systems. Progress should be judged not only by reference-genome accuracy, but by calibrated and experimentally validated generalization across plant diversity.

## Introduction

Deciphering how DNA sequence encodes gene regulation is a central challenge in plant biology. Cis-regulatory elements (CREs), including promoters, enhancers, silencers, and insulator-like elements, control transcription factor binding, chromatin state, transcription, and gene expression. Regulatory variation can influence developmental timing, environmental responses, and agronomic traits while preserving protein-coding function, making CREs promising targets for functional genomics and precision crop improvement (Wittkopp and Kalay, 2012; Swinnen et al., 2016; Cao et al., 2026a).

Seq2func models use machine learning, commonly deep neural networks, to map DNA sequence to molecular outputs such as chromatin accessibility, transcription factor occupancy, histone modifications, transcription initiation, RNA abundance, or chromatin interactions (Zhou and Troyanskaya, 2015; Kelley et al., 2016; Li et al., 2023). Here, “sequence-to-function (seq2func)” explicitly refers to computational models that map DNA sequence (and, when available, genomic context such as chromatin or haplotype annotations) to measurable regulatory outputs (e.g., chromatin accessibility, TF occupancy, histone marks, transcriptional activity, or chromatin interaction signals). In human and animal systems, these models have supported non-coding variant scoring, regulatory interpretation, and in silico sequence design (Gosai et al., 2024; Aksu and Vingron, 2026). Advances in telomere-to-telomere (T2T) genome assembly, graph-based pan-genome construction, and single-cell multi-omics are creating comparable opportunities in plants (Garg et al., 2024). However, plant genomes vary greatly in repeat content, ploidy, transposable-element composition, duplication, and structural variation (Cao et al., 2025, 2026; Kong et al., 2026). Regulatory output also depends on tissue, development, hormone state, biotic interactions, and environment. Evaluation based on randomly held-out loci from a reference genome therefore measures interpolation in a familiar setting rather than prediction of the variation most relevant to plant biology and breeding.

In this Mini Review, we examine the foundations and emerging applications of seq2func models in plant regulatory genomics. We discuss how pan-genomic variation, transposable elements, polyploidy, distal regulation, and context dependence challenge reference-based prediction; assess the complementary roles of observational profiling, reporter assays, and endogenous genome editing in moving from correlation toward causation; and propose biologically meaningful benchmarks for evaluating generalization. Finally, we outline a closed-loop framework combining context-aware modelling, uncertainty-guided perturbation, and continual learning to improve the discovery and validation of regulatory variants for climate-resilient crop improvement (**Figure 1**).

**Figure 1:**
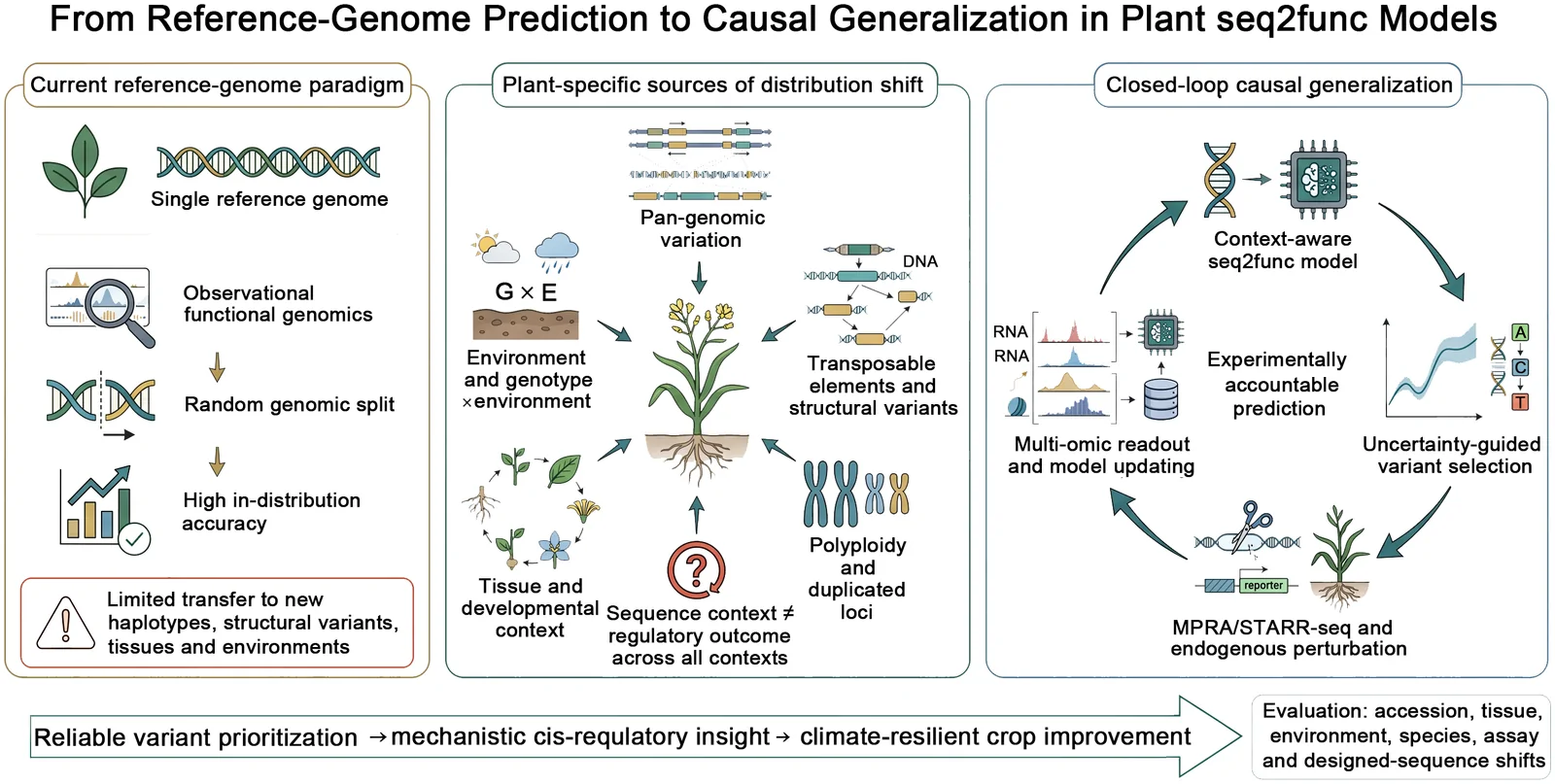
From reference-genome prediction to causal generalization in plant sequence-to-function models. Conventional models trained on observational data from a single reference genome can achieve high in-distribution performance but may fail under plant-relevant genetic and biological shifts. Pan-genomic variation, transposable elements, structural variation, polyploidy, tissue identity, development, and environmental responses alter the relationship between DNA sequence and regulatory activity. A closed-loop framework integrates context-aware seq2func modelling, uncertainty-guided candidate selection, massively parallel reporter assays (MPRAs) and self-transcribing active regulatory region sequencing (STARR-seq), endogenous perturbation, multi-omic readouts, and iterative model updating to enable experimentally accountable predictions for variant prioritization and crop improvement.

## Sequence-to-function models and plant regulatory genomics

Seq2func models approximate a mapping from DNA sequence to a measured regulatory output. Inputs are commonly one-hot-encoded DNA sequences, but may also include genotype, chromatin contacts, epigenomic measurements, or cell-state embeddings. Outputs can be binary labels, quantitative signal tracks, or gene-level measurements. Convolutional networks identify local motif-like patterns and provide a strong baseline for seq2func by learning regulatory “grammar” from local sequence context, whereas attention-based, state-space, and other long-context architectures can incorporate more distal sequence information (Kelley et al., 2016; Avsec et al., 2021). Long-context architectures can help represent distal dependencies that arise from enhancers, chromatin interactions, and repetitive sequence context. Long sequence windows may be important in plants, because regulatory effects can involve distal CREs, repetitive DNA, and chromatin architecture; however, longer context alone does not guarantee mechanistic insight or generalization. Instead, meaningful gains typically require appropriate architectural inductive bias, training diversity, and evaluation under biologically relevant distribution shifts.

Genomic language models provide self-supervised sequence representations, and several plant-specific or plant-applied foundation models have recently emerged, including PlantCaduceus, AgroNT, and plant-adapted nucleotide transformer architectures (Dalla-Torre et al., 2025; Liu et al., 2025; Morrell and Pakhomov, 2025; Mummadi et al., 2025; Zhai et al., 2025). In addition to foundation models, plant seq2func research also includes (i) promoter- and regulatory-element prediction models trained on plant chromatin accessibility or TF-occupancy readouts, (ii) Convolutional Neural Network (CNN)- style baselines trained directly on plant ATAC-seq profiles, (iii) Enformer/Borzoi-style long-range architectures adapted to plant sequence contexts, and (iv) plant variant-effect prediction benchmarks that test generalization across accessions and non-reference alleles/structural variants. Functional-genomics supervision remains important for learning direct sequence–activity relationships relevant to defined molecular phenotypes (Nagai et al., 2026). Extensive plant genome sequencing, including telomere-to-telomere assemblies, has generated resources for seq2func learning (Liu et al., 2024; Jiang et al., 2025; Xu et al., 2025). RNA-seq and nascent-transcription assays quantify transcriptional output; ATAC-seq and DNase-seq profile chromatin accessibility; ChIP-seq and CUT&Tag characterize transcription factor binding and histone modifications; and chromosome-conformation assays identify distal contacts (Buenrostro et al., 2013; Kaya-Okur et al., 2019). These assays provide complementary rather than interchangeable labels. Multitask learning may improve data efficiency across assays, tissues, genotypes, and species, but aggregate scores can conceal poor performance for tissue-specific, developmental, or stress-responsive programs.

Early studies demonstrate feasibility in plants. Peleke et al. (2024) developed interpretable models linking gene sequence to transcript abundance in *Arabidopsis thaliana*, *Solanum lycopersicum* (tomato), *Sorghum bicolor* (sorghum), and *Zea mays* (maize), identifying conserved and species-specific predictive features. Transfer was stronger between sorghum and maize than between more distant species (Peleke et al., 2024). In maize, DeepCBA integrated sequence, chromatin accessibility, and chromatin-interaction information to predict gene expression (Wang et al., 2024), illustrating the contribution of distal regulatory context. Together, these studies provide proof of principle while emphasizing the importance of chromatin, long-range regulation, and evolutionary diversity.

## Why plant genomes challenge seq2func prediction

### Pan-genomic diversity, transposable elements, and genome structure

A reference genome represents only one realization of a species’ regulatory sequence space. Plant pan-genomes reveal extensive variation in gene content and non-coding DNA, including promoter indels, inversions, copy-number changes, presence–absence variation, and transposable-element insertions (Danilevicz et al., 2020; Shi et al., 2023). Such variants can alter motif content, promoter architecture, enhancer–promoter distance, local chromatin, and genome organization. Maize provides a prominent example of extensive structural and transposable-element variation (Brunner et al., 2005; Hufford et al., 2021), but comparable diversity occurs in rice, soybean, wheat, tomato, and other crops (Wang et al., 2018; Walkowiak et al., 2020; Naik et al., 2025). Models trained on one reference accession may therefore fail on alleles relevant to breeding populations.

Transposable elements can contribute promoters, enhancers, transcription factor motifs, methylation targets, and chromatin-boundary effects (Chuong et al., 2017). Their impact depends on insertion position, sequence age, epigenetic state, neighboring elements, and biological context (Stuart et al., 2016). Repeat-rich genomes also increase the risk that models exploit homology or composition rather than transferable regulatory features (Whalen et al., 2022). Performance should therefore be reported separately for unique and repetitive sequences, reference and non-reference transposable-element insertions, and structurally altered loci. Graph genomes and haplotype-resolved assemblies could improve representation of alternate sequences, although most current seq2func architectures assume linear inputs (Danilevicz et al., 2020; Shi et al., 2023; Naik et al., 2025).

Plant CREs can also act far from their target genes. In maize, widespread long-range cis-regulatory elements highlight the importance of distal regulatory landscapes (Ricci et al., 2019). Models restricted to proximal promoters may omit relevant elements, whereas longer sequence windows increase computational costs and data requirements. Comparative studies are needed to determine when local, long-range, or multimodal models yield meaningful gains.

### Polyploidy and duplicated regulatory systems

Polyploidy is widespread in crops such as wheat, canola, cotton, and sugarcane. Hexaploid black nightshade (*Solanum nigrum*) further illustrates the value of haplotype-resolved assemblies for dissecting polyploid genome structure (Kong et al., 2026). Duplicate genes and subgenomes may retain, partition, or diversify regulatory functions; therefore, homeologous loci can be highly similar in sequence but differ in chromatin state, expression, or environmental responsiveness (Kong et al., 2026). In the hexaploid kiwifruit genome, short-read ATAC-seq, ChIP-seq, and RNA-seq reads can map ambiguously across homologous loci, potentially making distinct regulatory states appear similar (Li et al., 2025). In such settings, training labels (e.g., accessibility peaks or expression targets aggregated at a gene level) can inadvertently mix homeolog-specific regulatory signals, which may inflate aggregate performance while degrading homeolog-specific predictive fidelity. Evaluation should therefore quantify homeolog-specific activity, subgenome dominance, copy-number variation, and sensitivity to mapping ambiguity rather than only aggregate gene-level performance. In bread wheat, related regulatory sequences in the A, B, and D subgenomes can show subgenome-biased expression across tissues and environments (Ramírez-González et al., 2018). Evaluation should therefore quantify homeolog-specific activity, subgenome dominance, copy-number variation, and sensitivity to mapping ambiguity rather than only aggregate gene-level performance. Phased assemblies and homeolog-aware assays will be essential.

### Context dependence: tissues, development, and environment

DNA sequence defines regulatory potential, but output depends on the cellular and environmental context in which it is interpreted. Transcription factor abundance, cofactors, chromatin accessibility, DNA methylation, histone state, hormone signaling, and developmental history determine whether a motif or CRE is active. For example, Dehydration-Responsive Element-Binding/C-repeat Binding Factor (DREB/CBF) motifs may occur in both leaf and root promoters but activate transcription primarily during drought or cold stress, when relevant transcription factors accumulate and the locus becomes accessible (Yamaguchi-Shinozaki and Shinozaki, 2006). Likewise, the sequence of FLOWERING LOCUS C (FLC) remains unchanged during vernalization, but prolonged cold induces chromatin-based repression and reduced transcription (Angel et al., 2011). These examples represent a concept shift: the DNA input is unchanged, but the relationship between sequence and regulatory output differs among cell states or treatments. Genotype-by-environment interactions (G×E) add another layer of complexity. Natural accessions may carry similar drought-responsive motifs but differ in induction because of variation in ABA signaling, chromatin state, transcription factor abundance, or linked variants (Seymour et al., 2016). In maize and wheat, regulatory effects and homeolog expression biases can likewise change under heat, drought, or pathogen pressure (Lovell et al., 2021).

Context-aware models can incorporate tissue, developmental stage, treatment, chromatin profiles, or transcriptomic embeddings, enabling different predictions for the same sequence in different conditions. However, models trained under limited laboratory treatments may remain unreliable in fluctuating field environments, which is especially relevant for climate-resilient crop improvement. Studies should distinguish context-specific prediction in a defined biological setting from context-general prediction in an unobserved tissue, treatment, genotype, or field condition.

## From observational prediction to causal inference

Genome-wide functional-genomics assays provide essential maps of regulatory activity in endogenous chromatin. However, observational associations do not establish that a nucleotide, motif, or CRE causally determines a measured output. A motif may be functional, correlated with a functional feature, or associated with a broader genomic environment. Similarly, accessibility may cause transcription, result from transcription, or reflect another regulatory process. High held-out accuracy on observational data should not therefore be interpreted as evidence that a model has recovered causal regulatory rules.

MPRAs and STARR-seq allow systematic testing of natural and synthetic fragments. In tobacco (*Nicotiana tabacum*) leaves, STARR-seq identified plant enhancer activity and functional sequence features (Jores et al., 2020). Comparable MPRA platforms have been adapted to protoplasts and other plant systems (Cuperus et al., 2017). These assays efficiently test alleles, motif combinations, and designed sequences, but episomal constructs may not reproduce endogenous chromatin, methylation, genomic position, or long-range contacts. They should therefore be viewed as measurements of intrinsic or assay-specific regulatory potential.

CRISPR-based editing enables direct tests in native genomic contexts through deletions, promoter replacement, base editing, prime editing, and CRISPR interference or activation. Promoter editing in tomato has shown that targeted cis-regulatory modifications can create quantitative trait variation (Rodríguez-Leal et al., 2017). Similar approaches have been applied in maize and rice (Zhang et al., 2018). A practical strategy is hierarchical: use reporter assays for broad exploration, endogenous editing for causal validation, and multi-omic profiling to characterize molecular consequences.

### Bridging seq2func predictions to organismal phenotypes in plants

The review repeatedly invokes crop improvement, but the hardest step is often the connection from predicted regulatory activity to organismal phenotype. Seq2func scores can be integrated into downstream genetics and breeding pipelines. For example, expression-QTL integration, genome-wide association study (GWAS) fine-mapping, and genomic prediction models can use seq2func variant effect scores as features or priors to prioritize candidate functional variants. In stress-relevant settings, seq2func-derived scores can also support hypothesis-driven selection of regulatory elements whose predicted activity changes across environments. A practical question for future benchmarking is whether incorporating seq2func scores improves (i) GWAS credible set narrowing and fine-mapping calibration or (ii) genomic selection accuracy for traits with genotype-by-environment dependence in plants. Because plant phenotypes integrate multiple regulatory layers, these approaches should report performance gains relative to baseline polygenic models and should test robustness across accessions, tissues, and field-relevant environments.

### Evaluating generalization in plant seq2func models

Random locus-level splits within one reference genome estimate in-distribution performance, but do not test the settings in which plant models are often deployed. Benchmarking should include held-out accessions or haplotypes; structural variants such as promoter and transposable-element insertions, inversions, copy-number changes, and non-reference distal elements; unobserved tissues, cell types, developmental stages, and environmental treatments; assay shifts between genomic profiling, reporter assays, and endogenous perturbation; transfer across species; and designed variants or synthetic sequences.

These tests represent distinct distribution shifts (**Table 1**). These tests also motivate model-family-specific reporting: for covariate shift, both CNN-style motif learners and long-context/transformer-style models should be evaluated on non-reference sequence spaces; for label shift, models trained on different assay modalities should report how calibration transfers; and for concept shift, context-aware models should report stratified performance across tissues, developmental stages, and stress-relevant treatments. Covariate shift occurs when sequence distributions change, as for new haplotypes, structural variants, or synthetic promoters. Label shift occurs when the measurement process changes, such as between episomal reporter activity and endogenous chromatin activity. Concept shift occurs when tissues, development, or environments alter the mapping from sequence to activity. Models should report calibration and performance stratified by variant type, repeat class, chromatin context, genotype, tissue, treatment, and distance from target genes. Their domain of validity must be explicit, especially for breeding and genome-editing applications.

**Table 1:** Distribution shifts in plant seq2func benchmarking and corresponding test designs.

Shift type	Biological driver	Example test design	Relevant plant context	Representative seq2func model families (examples)
Covariate shift	Input sequence distribution changes	Train on reference accession; test on non-reference haplotypes, synthetic promoters, or TE insertions	Pan-genome variation, structural variants, engineered edits	Reference-trained sequence models (e.g., CNN/attention-based seq2func), and plant foundation models
Label shift	Measurement process or output definition changes	Compare model predictions across ATAC-seq, STARR-seq, and endogenous perturbation readouts	Episomal vs. native chromatin activity; assay platform differences	Assay-specific models trained on single-label modalities (ATAC-seq-only vs MPRA/STARR-seq-only)
Concept shift	Sequence–output relationship changes across contexts	Train on leaf chromatin data; test on root, seed, or drought-stressed tissue	Tissue specificity, developmental stage, environmental response	Context-aware multi-omics and long-context models trained with multi-tissue/stress augmentation

Interpretability methods, including attribution maps and in silico mutagenesis, can prioritize motifs and candidate variants but do not provide causal evidence. Interpretability methods, including attribution maps and in silico mutagenesis, should be treated as hypotheses rather than causal proof. Strong explanations should generate edit predictions that are prospectively validated in independent sequence and biological contexts. Stability across independently trained models should also be assessed. When attribution is trustworthy, it should recover known motifs with appropriate positional localization, remain consistent across training runs and model families, and agree with perturbation-based evidence under the same assay type. Major failure modes include attribution saturation (where importance becomes uniformly high), motif mislocalization (importance shifts away from the true functional site), and false-positive patterns driven by repetitive content or assay-specific biases. Accordingly, the review should explicitly report whether TF-MoDISco-style motif clustering and cross-model stability analysis were performed, and should recommend that predicted motif edits be prospectively validated before publication.

## Toward closed-loop plant regulatory AI

The combinatorial size of regulatory sequence space prevents exhaustive experimentation. Active learning can prioritize experiments that maximize expected information gain, including sequences with high uncertainty, model disagreement, underrepresented haplotypes, or strongly context-dependent predictions. Candidate sets may include natural alleles, motif edits, promoter replacements, transposable-element insertions, and synthetic combinations. Selection should balance uncertainty with sequence diversity, experimental feasibility, and crop relevance. Continual learning could then update models as perturbation data reveal systematic errors. Naïve fine-tuning risks catastrophic forgetting, whereas three complementary strategies, replay-based rehearsal of prior data, parameter regularization (e.g., elastic weight consolidation), and modular adapter architectures that add task-specific parameters without overwriting shared representations, may preserve earlier capabilities while incorporating new evidence (Kirkpatrick et al., 2017; Parisi et al., 2019). However, practical barriers are particularly severe in plants: data ownership and sharing constraints across institutions and breeding programs, the lack of standardized perturbation datasets across assays/labs, and long generation times and seasonal scheduling that limit iteration speed. Therefore, closed-loop continual learning in plants should be designed around delayed feedback, cross-lab dataset harmonization, and explicit uncertainty calibration under distribution shifts. This framework creates an iterative loop in which observations provide baseline knowledge, model uncertainty identifies informative experiments, perturbations test candidate mechanisms, and validated evidence improves predictive models.

Shared datasets and standards will be required. Useful benchmarks should integrate reference and pan-genome sequences, metadata-rich multi-omics, reporter measurements, endogenous perturbations, and predefined out-of-distribution tests. Metadata should specify genotype, haplotype, assembly, tissue, developmental stage, growth environment, treatment, assay, and replication. Anchor systems such as Arabidopsis, rice, maize, sorghum, tomato, soybean, and wheat can reveal which regulatory principles are conserved and which depend on lineage or genome architecture.

## Conclusions

Seq2func models offer a promising route to connect plant DNA sequence with molecular regulatory activity. Their value will depend not only on reference-genome accuracy but on generalization across genetic, genomic, developmental, and environmental diversity. Reference-based observational data remain indispensable, yet they cannot alone establish reliable causal predictions for non-reference alleles, structural variants, or engineered regulatory sequences.

Plant regulatory AI should therefore be evaluated across accessions, tissues, developmental stages, environments, and species; report calibrated uncertainty and domains of validity; and test mechanistic hypotheses through reporter assays and endogenous perturbation. Pan-genomes, context-aware modelling, active learning, and continual learning can support iterative systems in which experiments expose and correct predictive failures. A practical near-term roadmap is to establish two or three well-characterized anchor loci in a major crop, validated by both MPRA and endogenous editing across multiple accessions and environments, as community benchmarks for testing model generalization under defined distribution shifts. Treating seq2func models as experimentally accountable virtual assays, rather than static reference-genome annotation tools, could advance the discovery of cis-regulatory mechanisms and the prioritization of regulatory variants and genome edits for resilient crop improvement.

## References

[B1] Aksu E. D. Vingron M. (2026). Context-aware sequence-to-function model of human gene regulation. Nat. Commun. 17, 6200. doi: 10.1038/s41467-026-75527-2 42448699 PMC13370016

[B2] Angel A. Song J. Dean C. Howard M. (2011). A polycomb-based switch underlying quantitative epigenetic memory. Nature 476, 105–108. doi: 10.1038/nature10241 21785438

[B3] Avsec Ž. Agarwal V. Visentin D. Ledsam J. R. Grabska-Barwinska A. Taylor K. R. . (2021). Effective gene expression prediction from sequence by integrating long-range interactions. Nat. Methods 18, 1196–1203. doi: 10.1038/s41592-021-01252-x 34608324 PMC8490152

[B4] Brunner S. Fengler K. Morgante M. Tingey S. Rafalski A. (2005). Evolution of DNA sequence nonhomologies among maize inbreds. Plant Cell 17, 343–360. doi: 10.1105/tpc.104.025627 15659640 PMC548811

[B5] Buenrostro J. D. Giresi P. G. Zaba L. C. Chang H. Y. Greenleaf W. J. (2013). Transposition of native chromatin for fast and sensitive epigenomic profiling of open chromatin, DNA-binding proteins and nucleosome position. Nat. Methods 10, 1213–1218. doi: 10.1038/nmeth.2688 24097267 PMC3959825

[B6] Cao Y. Feng X. Ding B. Huo H. Abdullah M. Hong J. . (2025). Gap-free genome assemblies of two Pyrus bretschneideri cultivars and GWAS analyses identify a CCCH zinc finger protein as a key regulator of stone cell formation in pear fruit. Plant Commun. 6, 101238. doi: 10.1016/j.xplc.2024.101238 40071379 PMC11956113

[B7] Cao Y. Wen Z. He W. Liu Y. Yang J. Hong J. . (2026a). Overexpression of Camellia oleifera ColERF04 accelerates flowering in transgenic Arabidopsis. Ind. Crops Prod. 251, 124139. doi: 10.1016/j.indcrop.2026.12413942574925

[B8] Cao Y. Yang J. Liu Y. Song C. Wang L. Lin M. (2026b). Integrative genomics of Oil-Camellia: From multi-omics dissection of key traits to mechanism-informed molecular breeding. Ind. Crops Prod. 251, 124249. doi: 10.1016/j.indcrop.2026.12424942574925

[B9] Chuong E. B. Elde N. C. Feschotte C. (2017). Regulatory activities of transposable elements: from conflicts to benefits. Nat. Rev. Genet. 18, 71–86. doi: 10.1038/nrg.2016.139 27867194 PMC5498291

[B10] Cuperus J. T. Groves B. KuChina A. Rosenberg A. B. Jojic N. Fields S. . (2017). Deep learning of the regulatory grammar of yeast 5′ untranslated regions from 500,000 random sequences. Genome Res. 27, 2015–2024. doi: 10.1101/gr.224964.117 29097404 PMC5741052

[B11] Dalla-Torre H. Gonzalez L. Mendoza-Revilla J. Lopez Carranza N. Grzywaczewski A. H. Oteri F. . (2025). Nucleotide transformer: building and evaluating robust foundation models for human genomics. Nat. Methods 22, 287–297. doi: 10.1038/s41592-024-02523-z 39609566 PMC11810778

[B12] Danilevicz M. F. Fernandez C. G. T. Marsh J. I. Bayer P. E. Edwards D. (2020). Plant pangenomics: approaches, applications and advancements. Curr. Opin. Plant Biol. 54, 18–25. doi: 10.1016/j.pbi.2019.12.005 31982844

[B13] Garg V. Bohra A. Mascher M. Spannagl M. Xu X. Bevan M. W. . (2024). Unlocking plant genetics with telomere-to-telomere genome assemblies. Nat. Genet. 56, 1788–1799. doi: 10.1038/s41588-024-01830-7 39048791

[B14] Gosai S. J. Castro R. I. Fuentes N. Butts J. C. Mouri K. Alasoadura M. . (2024). Machine-guided design of cell-type-targeting cis-regulatory elements. Nature 634, 1211–1220. doi: 10.1038/s41586-024-08070-z 39443793 PMC11525185

[B15] Hufford M. B. Seetharam A. S. Woodhouse M. R. Chougule K. M. Ou S. Liu J. . (2021). De novo assembly, annotation, and comparative analysis of 26 diverse maize genomes. Science 373, 655–662. doi: 10.1126/science.abg5289 34353948 PMC8733867

[B16] Jiang L. Li X. Lyu K. Wang H. Li Z. Qi W. . (2025). Rosaceae phylogenomic studies provide insights into the evolution of new genes. Hortic. Plant J. 11, 389–405. doi: 10.1016/j.hpj.2024.02.00242574925

[B17] Jores T. Tonnies J. Dorrity M. W. Cuperus J. T. Fields S. Queitsch C. (2020). Identification of plant enhancers and their constituent elements by STARR-seq in tobacco leaves. Plant Cell 32, 2120–2131. doi: 10.1105/tpc.20.00155 32409318 PMC7346570

[B18] Kaya-Okur H. S. Wu S. J. Codomo C. A. Pledger E. S. Bryson T. D. Henikoff J. G. . (2019). CUT&Tag for efficient epigenomic profiling of small samples and single cells. Nat. Commun. 10, 1930. doi: 10.1038/s41467-019-09982-5 31036827 PMC6488672

[B19] Kelley D. R. Snoek J. Rinn J. L. (2016). Basset: learning the regulatory code of the accessible genome with deep convolutional neural networks. Genome Res. 26, 990–999. doi: 10.1101/gr.200535.115 27197224 PMC4937568

[B20] Kirkpatrick J. Pascanu R. Rabinowitz N. Veness J. Desjardins G. Rusu A. A. . (2017). Overcoming catastrophic forgetting in neural networks. Proc. Natl. Acad. Sci. 114, 3521–3526. doi: 10.1073/pnas.1611835114 28292907 PMC5380101

[B21] Kong B. Li X. Zhang Z. Luo W. Cao Y. (2026). Haplotype-resolved genome of hexaploid Solanum nigrum: Insights into origin, evolution, and steroidal glycoalkaloid biosynthesis. Cell Rep. 45, 117037. doi: 10.1016/j.celrep.2026.117037 41758653

[B22] Li X. Kuhl H. Zhu S. Xie S. Zhang C. Huo L. . (2025). The haplotype-resolved genome assembly of the hexaploid kiwifruit Actinidia deliciosa reveals its hybrid origin and polysomic inheritance. Plant Commun. 6, 101436. doi: 10.1016/j.xplc.2025.101436 40629692 PMC12545813

[B23] Li Z. Gao E. Zhou J. Han W. Xu X. Gao X. (2023). Applications of deep learning in understanding gene regulation. Cell Rep. Methods 3, 100384. doi: 10.1016/j.crmeth.2022.100384 36814848 PMC9939384

[B24] Liu J. Chen Z. Zhao Z. (2025). Assessing the accuracy of forest above-ground biomass and carbon storage estimation by meta-analysis based close-range remote sensing. Forestry Res. 5, e017. doi: 10.48130/forres-0025-0017 40969538 PMC12441908

[B25] Liu W. Liu C. Chen S. Wang M. Wang X. Yu Y. . (2024). A nearly gapless, highly contiguous reference genome for a doubled haploid line of Populus ussuriensis, enabling advanced genomic studies. Forestry Res. 4, e019. doi: 10.48130/forres-0024-0016 39524412 PMC11524312

[B26] Lovell J. T. Macqueen A. H. Mamidi S. Bonnette J. Jenkins J. Napier J. D. . (2021). Genomic mechanisms of climate adaptation in polyploid bioenergy switchgrass. Nature 590, 438–444. doi: 10.1038/s41586-020-03127-1 33505029 PMC7886653

[B27] Morrell P. L. Pakhomov S. V. (2025). Decoding nature’s grammar with DNA language models. Proc. Natl. Acad. Sci. 122, e2512889122. doi: 10.1073/pnas.2512889122 40658864 PMC12304990

[B28] Mummadi S. T. Islam M. K. Busov V. Wei H. (2025). Gene regulatory network prediction using machine learning, deep learning, and hybrid approaches. Forestry Res. 5, e014. doi: 10.48130/forres-0025-0014 40969532 PMC12441907

[B29] Naik Y. D. Rangari S. K. García-Caparros P. Jan F. Gangurde S. S. Zwart R. . (2025). “ Genomics, pan-genomics, and super pan-genomics of major oilseed crops,” in Breeding Climate Resilient and Future Ready Oilseed Crops ( Singapore: Springer), 7–41.

[B31a] Nagai T. Homma K. Kawamata Y. Yoshihara M. Kawakami E. Baba T. (2026). Leveraging large scale deep learning models for diagnosis and visual outcome prediction in retinitis pigmentosa. npj Digi Med. 9 (1), 137. 10.1038/s41746-025-02311-9 PMC12887015 41507439

[B30] Parisi G. I. Kemker R. Part J. L. Kanan C. Wermter S. (2019). Continual lifelong learning with neural networks: A review. Neural Networks 113, 54–71. doi: 10.1016/j.neunet.2019.01.012 30780045

[B31] Peleke F. F. Zumkeller S. M. Gültas M. Schmitt A. Szymański J. (2024). Deep learning the cis-regulatory code for gene expression in selected model plants. Nat. Commun. 15, 3488. doi: 10.1038/s41467-024-47744-0 38664394 PMC11045779

[B32] Ramírez-González R. H. Borrill P. Lang D. Harrington S. A. Brinton J. Venturini L. . (2018). The transcriptional landscape of polyploid wheat. Science 361, eaar6089. doi: 10.1126/science.aar6089 30115782

[B33] Ricci W. A. Lu Z. Ji L. Marand A. P. Ethridge C. L. Murphy N. G. . (2019). Widespread long-range cis-regulatory elements in the maize genome. Nat. Plants 5, 1237–1249. doi: 10.1038/s41477-019-0547-0 31740773 PMC6904520

[B34] Rodríguez-Leal D. Lemmon Z. H. Man J. Bartlett M. E. Lippman Z. B. (2017). Engineering quantitative trait variation for crop improvement by genome editing. Cell 171, 470–480. doi: 10.1016/j.cell.2017.08.030 28919077

[B35] Seymour D. K. Chae E. Grimm D. G. Martin Pizarro C. Habring-Müller A. Vasseur F. . (2016). Genetic architecture of nonadditive inheritance in Arabidopsis thaliana hybrids. Proc. Natl. Acad. Sci. 113, E7317–E7326. doi: 10.1073/pnas.1615268113 27803326 PMC5135357

[B36] Shi J. Tian Z. Lai J. Huang X. (2023). Plant pan-genomics and its applications. Mol. Plant 16, 168–186. doi: 10.1016/j.molp.2022.12.009 36523157

[B37] Stuart T. Eichten S. R. Cahn J. Karpievitch Y. V. Borevitz J. O. Lister R. (2016). Population scale mapping of transposable element diversity reveals links to gene regulation and epigenomic variation. elife 5, e20777. doi: 10.7554/elife.20777 27911260 PMC5167521

[B38] Swinnen G. Goossens A. Pauwels L. (2016). Lessons from domestication: targeting cis-regulatory elements for crop improvement. Trends Plant Sci. 21, 506–515. doi: 10.1016/j.tplants.2019.09.004 26876195

[B39] Walkowiak S. Gao L. Monat C. Haberer G. Kassa M. T. Brinton J. . (2020). Multiple wheat genomes reveal global variation in modern breeding. Nature 588, 277–283. doi: 10.1038/s41586-020-2961-x 33239791 PMC7759465

[B40] Wang W. Mauleon R. Hu Z. Chebotarov D. Tai S. Wu Z. . (2018). Genomic variation in 3,010 diverse accessions of Asian cultivated rice. Nature 557, 43–49. doi: 10.1038/s41586-018-0063-9 29695866 PMC6784863

[B41] Wang Z. Peng Y. Li J. Li J. Yuan H. Yang S. . (2024). DeepCBA: A deep learning framework for gene expression prediction in maize based on DNA sequences and chromatin interactions. Plant Commun. 5, 100985. doi: 10.1016/j.xplc.2024.100985 38859587 PMC11413363

[B42] Whalen S. Schreiber J. Noble W. S. Pollard K. S. (2022). Navigating the pitfalls of applying machine learning in genomics. Nat. Rev. Genet. 23, 169–181. doi: 10.1038/s41576-021-00434-9 34837041

[B43] Wittkopp P. J. Kalay G. (2012). Cis-regulatory elements: molecular mechanisms and evolutionary processes underlying divergence. Nat. Rev. Genet. 13, 59–71. doi: 10.1038/nrg3095 22143240

[B44] Xu C. Song L. Y. Li J. Zhang L. D. Guo Z. J. Ma D. N. . (2025). MangroveDB: A comprehensive online database for mangroves based on multi‐omics data. Plant Cell Environ. 48, 2950–2962. doi: 10.1111/pce.15318 39660842

[B45] Yamaguchi-Shinozaki K. Shinozaki K. (2006). Transcriptional regulatory networks in cellular responses and tolerance to dehydration and cold stresses. Annu. Rev. Plant Biol. 57, 781–803. doi: 10.1146/annurev.arplant.57.032905.105444 16669782

[B46] Zhai J. Gokaslan A. Schiff Y. Berthel A. Liu Z.-Y. Lai W.-Y. . (2025). Cross-species modeling of plant genomes at single-nucleotide resolution using a pretrained DNA language model. Proc. Natl. Acad. Sci. 122, e2421738122. doi: 10.1073/pnas.2421738122 40489624 PMC12184517

[B47] Zhang H. Si X. Ji X. Fan R. Liu J. Chen K. . (2018). Genome editing of upstream open reading frames enables translational control in plants. Nat. Biotechnol. 36, 894–898. doi: 10.1038/nbt.4202 30080209

[B48] Zhou J. Troyanskaya O. G. (2015). Predicting effects of noncoding variants with deep learning–based sequence model. Nat. Methods 12, 931–934. doi: 10.1038/nmeth.3547 26301843 PMC4768299

